# Assisted Reproductive Technology: A Ray of Hope for Infertility

**DOI:** 10.1021/acsomega.5c01643

**Published:** 2025-05-23

**Authors:** Rumiana Tenchov, Qiongqiong Angela Zhou

**Affiliations:** 2645 CAS, A division of the American Chemical Society, Columbus Ohio 43210, United States

## Abstract

Assisted reproductive technologies (ART) have revolutionized the field of reproductive medicine, offering hope to millions of individuals and couples facing fertility problems. From artificial insemination to cutting-edge gene editing techniques, ART enhances reproductive efficiency, transforming the landscape of reproductive medicine. While established techniques such as *in vitro* fertilization and intracytoplasmic sperm injection are currently widely used, emerging technologies such as *in vitro* gametogenesis, gene therapies, and stem cell-based therapies are expanding the boundaries of what is possible. ART is a rapidly advancing field; however, the application of certain novel and emerging technologies in humans is still highly experimental, tightly regulated, and surrounded by ethical and legal challenges. Many innovations in ART are currently being tested only on animal models, yet some have successfully transitioned to human applications, including preimplantation genetic testing, mitochondrial replacement therapy, laser-assisted hatching, time-lapse imaging, and *in vitro* maturation. Furthermore, artificial intelligence is transforming reproductive medicine by enabling precise embryo selection, optimizing clinical protocols, and predicting treatment outcomes. This report explores data from the CAS Content Collection to outline the research progress in ART, to identify key emerging concepts and challenges, and its societal impact in an effort to understand how ART continues to shape the future of reproductive healthcare. The novelty and merit of the article stem from the extensive, wide-ranging coverage of up-to-date scientific information accumulated in the CAS Content Collection, allowing for a unique, unmatched breadth of landscape analysis and in-depth insights.

## Graphical abstract

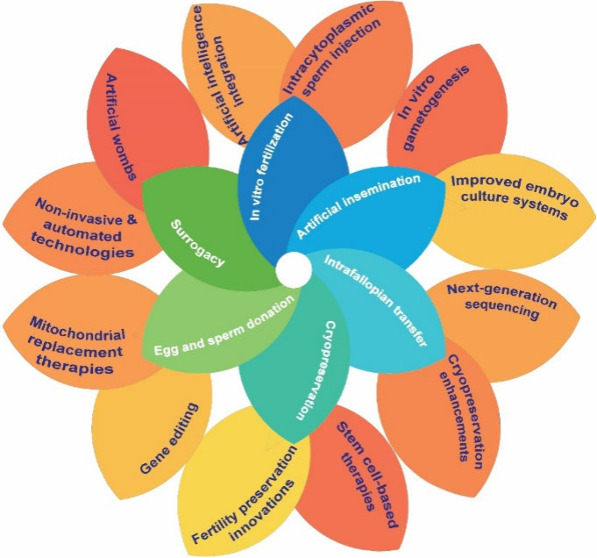

## Introduction

Assisted reproductive technology (ART) encompasses a broad spectrum of medical techniques designed to aid individuals and couples in overcoming infertility challenges, enabling the conception of a child.^1–9^ As infertility affects approximately 10–15% of couples worldwide, ART is a critical component of modern healthcare.^10,11^ Since its start with the birth of the first *in vitro* fertilization (IVF) baby in 1978,^12^ ART has evolved significantly, incorporating groundbreaking scientific and technological advancements. These developments have transformed the field of reproductive medicine, offering innovative solutions for diverse reproductive issues and expanding possibilities for parenthood.

ART procedures typically involve the handling of eggs, sperm, and embryos to achieve fertilization and implantation. Techniques, such as IVF and cryopreservation, are now standard practices in fertility clinics worldwide. In recent years, emerging technologies such as artificial intelligence (AI), genetic testing, and stem cell research have further refined ART, enhancing its success rates while addressing ethical and social implications. Furthermore, experimental innovations like *in vitro* gametogenesis (IVG) hold the promise of providing gametes for individuals who are unable to produce their own, potentially revolutionizing reproductive options for individuals with infertility. Noteworthy, ART is a rapidly advancing field, but the application of certain novel and emerging technologies in humans is still highly experimental, tightly regulated, and surrounded by various ethical and legal challenges. They rely heavily on animal models, which offer valuable insights into reproductive biology and the effects of various ART interventions.^13–15^

Along with advances and recent success in ART, certain major challenges and concerns exist. These include scientific hurdles such as efficiently replicating the complex microenvironment of the gonads in vitro; ensuring the genetic and epigenetic stability of laboratory-generated gametes; and achieving successful fertilization, implantation, and development using IVG-derived gametes, to mention a few. Important ethical considerations involve: (i) safety—risks of creating embryos from lab-generated gametes are unknown; (ii) designer babies—potential misuse for nontherapeutic genetic modifications; (iii) embryo overproduction—generating surplus embryos raises ethical concerns about their fate; (iv) consent and access—determining ownership and rights over iPSC-derived gametes. Furthermore, regulatory and social acceptance present additional challenges related to ART, for example, public perceptions and cultural attitudes toward creating gametes in the lab could pose serious barriers.

In this report, we explore data from the CAS Content Collection,^16^ the largest human-curated repository of scientific information, to outline the research progress in ART. We analyze the publication landscape to offer perspective into the latest advancements, to identify key emerging concepts and challenges associated with ART. We review the most discussed and emerging concepts and assess the strategies to improve ART. We first explore the traditional methods used in ART, with their advantages and shortcomings, then review the recent advancements providing novel options and improving success rates. The major types of substance classes commonly associated with ART have been characterized. The insights from the CAS Content Collection allowed us to identify *in vitro* fertilization and embryo transfer as the best and most widely explored areas in the field. Furthermore, the fastest growing promising novel methods in ART have been identified as artificial intelligence integration and *in vitro* gametogenesis. Special attention has been devoted to the ethical considerations associated with ART. By exploring its scientific basis, clinical applications, and societal impact, the report aims to provide a comprehensive understanding of how ART continues to shape the future of reproductive healthcare. The merit of the article stems from the extensive, wide-ranging coverage of the most up-to-date scientific information, allowing extensive breadth of landscape analysis and in-depth insights.

## CAS Content Collection Landscape

Our search in the CAS Content Collection^16^ for ART-related documents retrieved over 50,000 scientific publications (mostly journal articles and patents) for the period 2000–2024. The number of related documents has consistently grown over the last two decades, more than tripling in that time (Figure 1A). Reflecting the early success of *in vitro* fertilization, form the 1980 the number of ART-related research has exhibited exponential growth (Figure 1A, inset). Figure 1B summarizes the top patent offices with the most ART-associated patents. The World Intellectual Property Organization (WIPO) and the China patent office are notable leaders.

**Figure 1:**
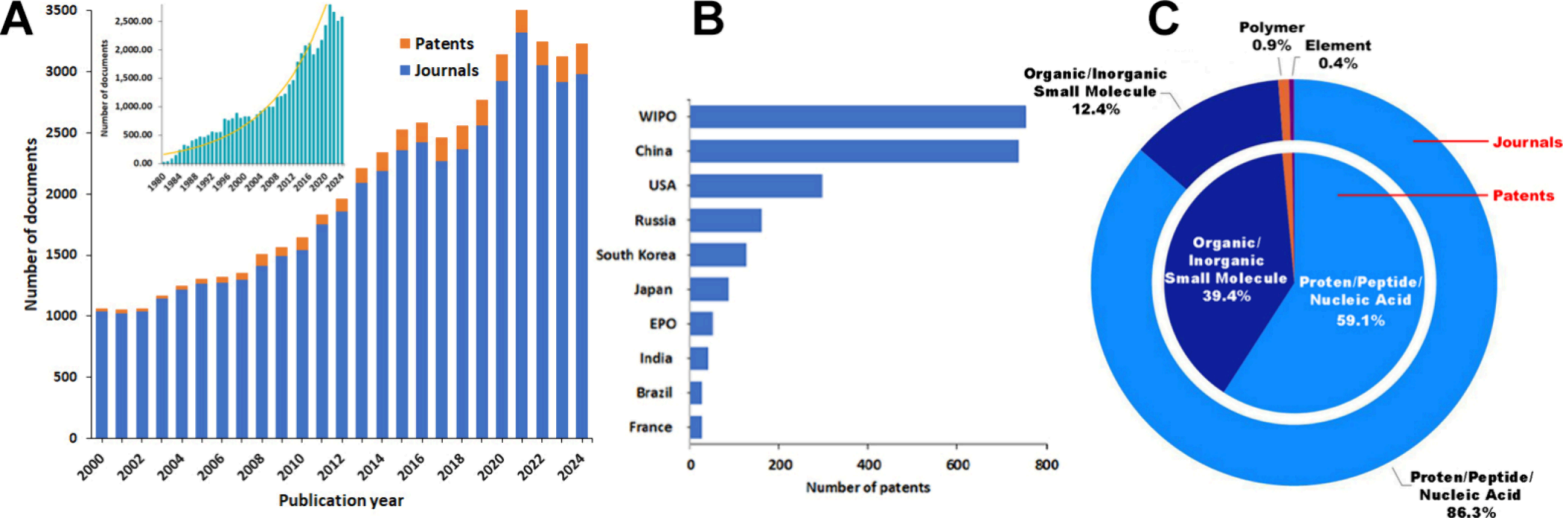
(A) Number of documents (journal articles and patents) related to ART in the CAS Content Collection for years 2000–2024. Inset: Document yearly growth from year 1980, with an exponential growth trendline. (B) Top patent offices with patents related to ART. (C) Distribution of substances associated with ART in journal (outer donut chart) and patent (inner pie chart) publications, broken down by substance class. Data from the CAS Content Collection for the period 2000–2024.

We surveyed the substance data extracted from the CAS REGISTRY^17^ regarding the types of substance classes commonly associated with ART. Our analysis indicates that proteins/peptides/nucleic acids and small molecules are most commonly associated with ART (Figure 1C). In patents, proteins/peptides/nucleic acids represent ∼60%, and in journals, ∼86% of publications. Small molecules are the second largest group, with 12% in journals and ∼40% in patents (Figure 1C).

Indeed, proteins, peptides, and nucleic acids play crucial roles in advancing ART. Their applications are expanding with the development of emerging biotechnological innovations. Exemplary specific roles of proteins, peptides, and nucleic acids in the emerging ART are described below:

### Proteins

(i) Growth factors and cytokines: proteins like bone morphogenetic proteins (BMP), insulin-like growth factor (IGF), and epidermal growth factor (EGF) improve oocyte maturation, sperm motility, and embryo development in culture media;^18,19^ antiapoptotic proteins (e.g., BCL-2) enhance embryo survival.^20,21^ (ii) Hormones and receptors: follicle-stimulating hormone (FSH), luteinizing hormone (LH), and hCG are used for ovarian stimulation in IVF;^22,23^ zona pellucida proteins (ZP1–4) are critical for sperm-egg binding and fertilization;^24,25^ albumin and serum proteins are used in culture media to stabilize embryos and prevent oxidative stress.^26–28^

### Peptides

(i) Synthetic peptides for sperm activation: CatSper channel-activating peptides can enhance sperm motility for ICSI.^29^ (ii) Antimicrobial peptides (AMPs) are used to prevent bacterial contamination in semen extenders and embryo culture media.^30,31^ (iii) Cell-penetrating peptides (CPPs) deliver gene-editing tools (CRISPR-Cas9) or protective molecules (e.g., antioxidants) into gametes/embryos.^32,33^

### Nucleic Acids

DNA/RNA analysis for genetic screening: (i) Preimplantation genetic testing (PGT-A/PGT-M) using PCR and NGS to screen embryos for aneuploidy or genetic disorders. Sperm RNA profiling helps identify male infertility biomarkers. (ii) Gene editing (CRISPR-Cas9) corrects mutations in embryos (e.g., mitochondrial DNA diseases); potential use in synthetic embryos or gametes from stem cells. (iii) Noncoding RNAs (miRNAs, lncRNAs): miRNAs regulate oocyte maturation and embryo implantation; exosomal RNAs in seminal fluid influence embryo development.^34–36^

### Emerging Trends

(i) Synthetic proteins/peptides: Custom-designed molecules to improve gamete quality and embryo viability. (ii) Nucleic acid therapeutics: mRNA-based treatments to enhance endometrial receptivity. (iii) Exosome-based therapies: using extracellular vesicles carrying proteins/nucleic acids to improve reproductive outcomes. Proteins, peptides, and nucleic acids are revolutionizing ART by enhancing the fertilization efficiency, embryo quality, and genetic safety. Future advances may include personalized reproductive medicine using these biomolecules.

### Small Molecules

Small molecules are the second largest group of substances represented in the ART-related documents, with 12% in journals and ∼40% in patents. They play several critical roles in the emerging trends of ART, enhancing efficiency, safety, and success rates. Small molecules (typically <900 Da) are revolutionizing ART by improving gamete quality, embryo viability, and implantation success while enabling cutting-edge techniques like IVG, stem cell-based reproduction, and personalized fertility treatments.^37–41^ Their role will expand further with advances in precision reproductive medicine. Table 1 summarizes the roles of small molecules in emerging ART.

**Table 1:** Roles of Small Molecules in Emerging ART

Application in ART	Exemplary small molecules used	Mechanism of action	Impact on ART
Oocyte maturation (IVM)	Forskolin, IBMX, melatonin, resveratrol	Modulate cAMP, reduce oxidative stress	Improves oocyte quality and meiotic competence
Sperm motility and capacitation	Caffeine, pentoxifylline, progesterone analogs	Enhance cAMP, Ca^2+^ signaling	Boosts sperm motility and fertilization rates (ICSI/IVF)
Embryo culture optimization	Rapamycin, scriptaid, l-carnitine	Inhibit mTOR/HDACs, reduce ROS	Enhances blastocyst formation and embryo viability
Endometrial receptivity	VEGF stimulators, dydrogesterone	Promote angiogenesis, mimic progesterone effects	Improves implantation success in FET cycles
In vitro gametogenesis (IVG)	Retinoic acid, BMP4	Induce germ cell differentiation from iPSCs	Enables lab-grown gametes for infertility treatments
Cryopreservation	Trehalose, DMSO alternatives	Stabilize cell membranes, prevent ice crystal formation	Increases survival of frozen oocytes/embryos
Epigenetic modulation	5-Azacytidine, valproic acid	DNMT/HDAC inhibition, correct imprinting errors	Reduces epigenetic defects in embryos
Nonhormonal ovarian stimulation	Letrozole, FSH receptor modulators	Aromatase inhibition, FSH pathway activation	Safer, personalized ovarian stimulation
Mitochondrial enhancement	CoQ10, MitoQ	Boost ATP production, reduce oxidative damage	Reverses age-related oocyte decline
3D bioprinting and organoids	Growth factor mimetics (e.g., BMPs)	Guide follicle/testicular tissue assembly	Future fertility restoration (e.g., artificial ovaries)

Thus, key trends enabled by small molecules include: (i) precision fertility—targeted modulation of gamete/embryo quality; (ii) stem cell-based reproduction—lab-generated gametes (IVG); (iii) reduced hormonal dependence—safer stimulation protocols; (iv) epigenetic safety—mitigating ART-induced epigenetic risks; (v) cryopreservation advances—higher post-thaw survival rates.

### Polymers

Polymers, represented by ∼1% in both patents and journal articles related to the field, also play roles in ART by improving biocompatibility, structural support, drug delivery, and cryopreservation. Their versatility enables advances in embryo culture, gamete storage, bioengineered reproductive tissues, and minimally invasive procedures.^42–44^ For example, polymers like hyaluronic acid and PEG improve biocompatibility by reducing immune rejection; alginates and collagen provide mechanical support by mimicking ECM for 3D culture; PLGA and chitosan nanoparticles provide controlled release for slow hormone/drug delivery; PVA and trehalose polymers play a role in cryoprotection, preventing freeze damage; fibrin and poloxamer gels play a role in bioadhesion, improving embryo transfer success,

## Assisted Reproductive Technology: Traditional Methods

Efforts to overcome infertility have a long history, from the first documented case of artificial insemination in 1790 by John Hunter in England,^45^ through the discovery of the hormonal control of ovulation that laid the groundwork for ovarian stimulation in ART,^46^ and the introduction of cryopreservation techniques for sperm^47^ in the 1950s, further with the development of *in vitro* techniques to study fertilization in mammals,^48,49^ as well as the research on ovarian stimulation and egg retrieval^46^ in the 1960s. The first pregnancy achieved through *in vitro* human fertilization of a human oocyte was reported in 1973 although it ended in miscarriage.^50^ It was not until 1978 that the first successful IVF pregnancy and live birth occurred,^51,52^ with the IVF becoming mainstream in the 1980s.^53,54^

Currently, the traditional methods of ART involve established and widely used techniques that have formed the foundation of infertility treatments (Figure 2). These methods primarily focus on the manipulation of eggs, sperm, and embryos to enhance the chances of conception.

**Figure 2:**
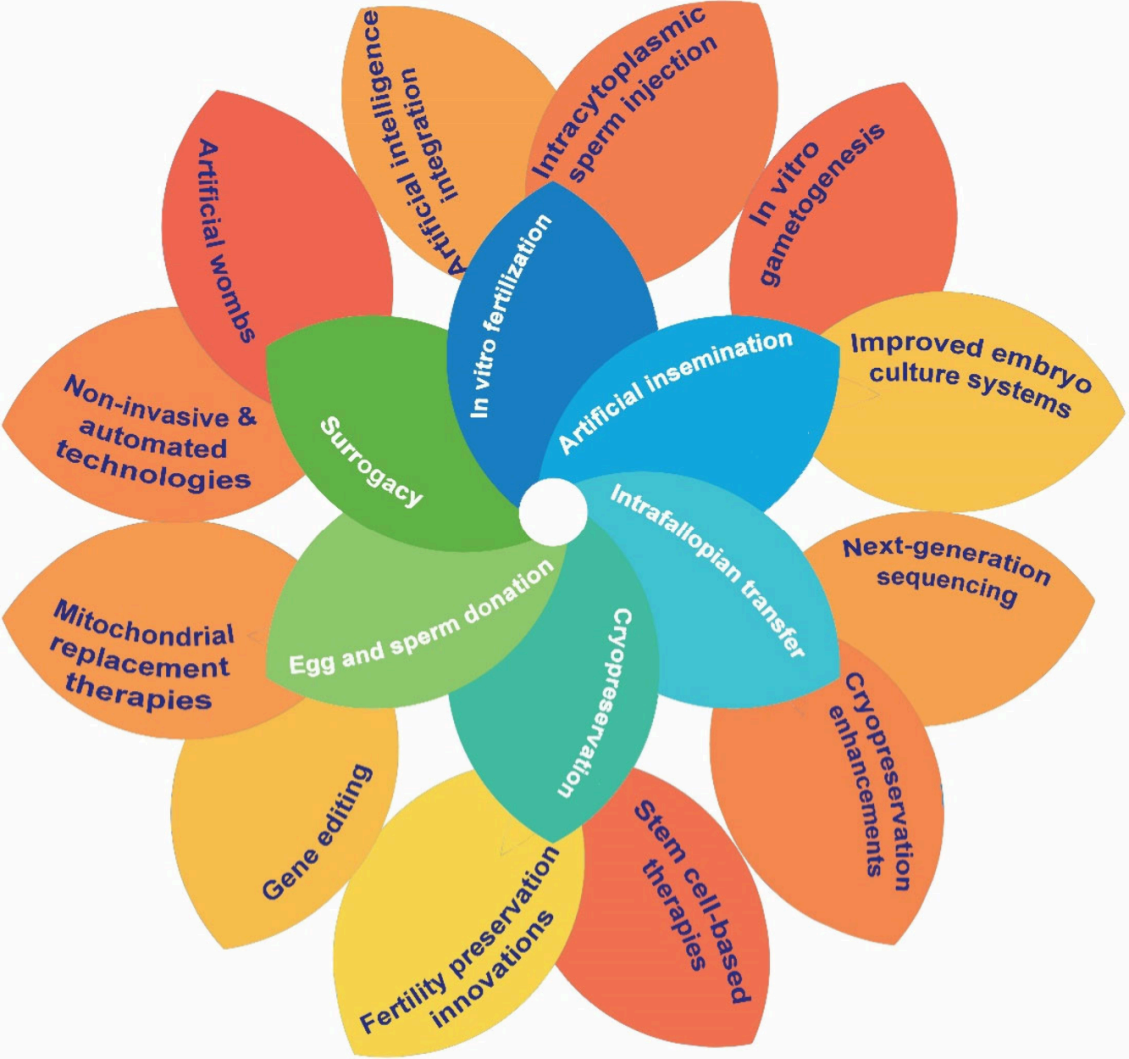
Traditional ART methods (inner blue-green circle) and recent advancements (outer yellow-orange circle).

### *In Vitro* Fertilization (IVF)

*In vitro* fertilization (IVF) is the most well-known ART procedure. It involves a process of fertilization, in which an egg is combined with sperm *in vitro*. IVF includes the steps of ovarian stimulation using fertility drugs to produce multiple eggs, retrieval of mature eggs through a minor surgical procedure, fertilization of eggs with sperm in a laboratory dish, and transfer of resulting embryos into the uterus. Currently fully integrated into clinical practice, it is successfully applied in tubal factor infertility, endometriosis, male factor infertility, and unexplained infertility. IVF is now a cornerstone of human fertility treatment, enabling millions of births worldwide. Success rates vary by age, with the highest success rates (30–40% per cycle) for women under 35. Rates decline significantly after age 40.^55–57^

### Artificial Insemination

Artificial insemination is a medical procedure in which sperm is introduced into a woman’s reproductive tract to facilitate fertilization and pregnancy. A sperm sample is collected, washed, and concentrated to isolate healthy sperm and then placed directly into the uterus (intrauterine insemination, IUI) or cervix (intracervical insemination, ICI) during ovulation. It is mainly applied in cases of mild male infertility, unexplained infertility, and cervical mucus issues. It is simpler and less invasive than IVF. Success rates are typically 10–20% per cycle, depending on factors like age and sperm quality.^58,59^

### Gamete Intrafallopian Transfer (GIFT)

Gamete intrafallopian transfer (GIFT) is a procedure that helps women conceive by placing eggs and sperm directly into the fallopian tubes. Eggs and sperm are collected and mixed before being placed into the fallopian tube via laparoscopy, allowing fertilization to occur naturally in the body. Used when one fallopian tube is functioning and there are no significant sperm issues. Requires a surgical procedure and general anesthesia. In contrast to IVF, which places fertilized eggs directly into the uterus, the GIFT technique allowed the eggs to fertilize and develop in the fallopian tube and then find their way to the uterus for implantation. It is less commonly used today due to advances in IVF.^60–62^

### Zygote Intrafallopian Transfer (ZIFT)

Zygote intrafallopian transfer (ZIFT) is similar to IVF, but the fertilized egg (zygote) is transferred into the fallopian tube instead of into the uterus. It is applied for patients with infertility but healthy fallopian tubes. Allows the zygote to develop in the natural environment of the fallopian tube. Combines the benefits of IVF and GIFT, but is less common now.^63,64^

### Cryopreservation (Fertility Preservation)

Cryopreservation (fertility preservation) involves freezing and storing reproductive cells, such as eggs, sperm, and embryos, for future use. Cryopreservation is now a routine procedure for embryos and sperm, and is becoming more common for oocytes. It is applied for fertility preservation, e.g., for cancer patients undergoing chemotherapy or radiation, with excess embryos from IVF, or delaying childbearing for personal or professional reasons. Vitrification (rapid freezing) has significantly improved outcomes compared to slow freezing. Success rates depend on the age at which eggs or sperm are frozen. Long-term storage costs can be significant.^65–67^

### Egg Donation and Sperm Donation

Egg donation and sperm donation can help people have children when they are not able to produce healthy eggs or sperm on their own. Eggs or sperm are donated by a third party and used in ART procedures such as IVF or intrauterine insemination to achieve pregnancy. Applied for individuals unable to produce viable gametes, such as women with premature ovarian failure or poor egg quality or men with no viable sperm. Widely used by older women, same-sex couples, and single parents. Donors are screened for medical and genetic conditions. Legal and ethical issues around donor anonymity and parental rights vary by country.^68,69^

### Surrogacy

Surrogacy involves a woman carrying and giving birth to a child for another person or couple using their embryos (gestational surrogacy) or their own egg (traditional surrogacy). While in traditional surrogacy the surrogate’s egg is fertilized with sperm (via IUI or IVF), making her the biological mother, in gestational surrogacy the surrogate carries an embryo created through IVF using the intended parents’ or donors’ eggs and sperm, so she has no genetic link to the child. Applied for individuals with uterine issues or medical conditions preventing pregnancy, also for same-sex male couples or single men. Surrogacy laws vary widely by country and region.^70,71^

Advantages of the traditional ART methods described above include: (i) proven track record including decades of successful use and refinement; (ii) customization—can be tailored to specific infertility causes; (iii) wide availability, offered by most fertility clinics worldwide. Traditional ART methods remain the backbone of modern infertility treatment, with ongoing advancements improving success rates and patient experiences. Still, traditional ART methods, while groundbreaking and beneficial for many, do have some shortcomings such as high costs, emotional and physical stress, lower success rates with age, risk of multiple births, and health risks including ovarian hyperstimulation syndrome as well as certain ethical and legal Issues. Recent advancements in ART are actively addressing these key shortcomings, directly tackling the cost, emotional strain, and physical demands of traditional methods. While challenges remain, innovations like AI, simplified protocols, and gentler procedures are making fertility treatments more efficient and patient-centric. For example, automation and AI in IVF laboratories, including AI-driven embryo selection (e.g., time-lapse imaging and machine learning) reduces failed cycles by picking the best-quality embryos, cutting repeat IVF costs; robotic ICSI improves precision, lowering lab costs over time. Next-generation sequencing (NGS) for PGT is now faster and more affordable, reducing the costs of failed implantations due to chromosomal abnormalities. PGT-A (preimplantation genetic testing for aneuploidy) improves live birth rates per transfer, reducing the emotional toll from repeated failures. Endometrial receptivity analysis (ERA) ensures that embryos are transferred at the optimal time. Oral ovulation stimulants (e.g., Letrozole, Clomiphene) are replacing some injectables, thus reducing physical burden. Also, long-acting FSH analogs (e.g., Corifollitropin alfa) require fewer injections.

## Recent Advancements in ART

Recent advancements in ART are transforming fertility treatments, providing more options, and improving success rates.

### Artificial Intelligence in ART

Artificial Intelligence (AI) is increasingly being integrated into ART to enhance efficiency, precision, and outcomes.^72–78^ It has been successfully utilized in several areas.

For **embryo selection**, AI algorithms analyze embryo images to assess their quality and potential for successful implantation.^73,79^ These algorithms use: (i) time-lapse imaging: AI monitors embryo development over time, evaluating factors such as morphology, cell division patterns, and dynamics; (ii) morphokinetic data: algorithms predict the likelihood of an embryo developing into a viable pregnancy by identifying subtle features not visible to the human eye. AI also helps improve **sperm selection** by (i) sperm motility analysis, identifying the most motile and morphologically normal sperm; (ii) DNA integrity checks, assessing DNA fragmentation levels in sperm to select the healthiest candidates.^80,81^

Machine learning models analyze multiple data points to **predict the success rate of IVF**, including patient history (age, hormonal levels, lifestyle factors), clinical data (ovarian reserve markers, endometrial receptivity), and embryo quality metrics. AI can **optimize ovarian stimulation protocols** by personalizing medication dosages based on patient-specific responses, and predicting ovarian response to stimulation, reducing the risk of ovarian hyperstimulation syndrome (OHSS).^82^

AI-driven **automation** streamlines processes in ART laboratories, including monitoring and controlling incubator conditions, standardizing embryo grading to minimize human error, and managing cryopreservation protocols.^76,77^ AI leverages **large data sets** from clinics and research studies to identify trends and factors influencing ART success and improve treatment protocols by recognizing patterns in patient and embryo data.^72^

AI is playing a transformative role in **reducing costs** in artificial reproduction techniques (ART), addressing one of the biggest barriers to accessibility. The major way for reducing costs is via **AI-driven efficiency**:

- Smarter embryo selection (biggest cost-saver): Time-lapse imaging + deep learning (e.g., EmbryoScope, LifeWhisperer^83–86^ ) predicts embryo viability with >90% accuracy, reducing failed transfers. Cost impact: fewer IVF cycles needed per live birth.
- Optimized ovarian stimulation: Algorithms (e.g., IVF2.0, Alife) personalize drug doses based on patient data (AMH, BMI, age), minimizing wasted medications.^87,88^ Cost impact: reduces medication costs.
- Automated sperm analysis: Tools like YO Sperm Analyzer or MobileHome^89–91^ provide instant, accurate sperm motility/morphology readings. Cost impact: cuts lab fees for basic diagnostics.

**Lowering emotional and physical burden** (indirect cost savings): (i) AI’s improved embryo/sperm selection reduces psychological toll and financial strain from multiple IVF attempts; (ii) AI models (e.g., Fairtility’s CHLOE^92^) predict optimal protocols per patient, avoiding costly trial-and-error approaches.

While not yet universal, AI adoption in fertility clinics is making treatments more affordable and efficient. In the near future, AI could democratize access to ART by slashing costs by 30–50% for many patients.

### In Vitro Gametogenesis (IVG)

IVG represents a groundbreaking advancement in the field of ART offering new possibilities for addressing infertility, understanding human reproduction, and exploring genetic disorders.^93–96^ IVG is an experimental technology that enables the creation of sperm or eggs from somatic cells such as skin or blood cells. IVG involves the differentiation of pluripotent stem cells (PSCs), such as embryonic stem cells (ESCs) or induced pluripotent stem cells (iPSCs), into gametes. This process mimics the natural progression of gametogenesis, where primordial germ cells develop into mature gametes through intricate molecular and cellular pathways. Researchers have successfully produced functional gametes in animal models such as mice, leading to healthy offspring. In 2024, scientists at Kyoto University created precursors to human gametes from induced pluripotent stem cells (iPSCs).^97^

While IVG has been successfully demonstrated in animal models, translating these techniques to human systems remains a work in progress due to the complexity of human gametogenesis and important ethical concerns. Potential uses of IVG include: providing gametes for individuals unable to produce viable eggs or sperm; enabling same-sex couples to have genetically related children; and addressing infertility due to age or medical conditions. IVG can be used to study early embryonic development and genetic diseases in controlled environments. There are ethical concerns regarding embryo creation and manipulation—it might lead to ethical dilemmas about creating and discarding large numbers of embryos, etc.^98,99^ Safety and efficacy need extensive validation before clinical application. One of the primary safety concern regarding germline editing is the lack of sufficient data on long-term consequences and potential off-target effects.^100^ There is a growing emphasis on involving the public in discussions about the ethical, legal, and social implications of genetic material editing.

### Stem Cell-Based Therapies

Stem cell-based therapies have emerged as a promising avenue in ART, leveraging the regenerative and differentiation potential of stem cells to enhance reproductive outcomes. It is paving the way for advanced reproductive treatments.^101–106^ The application of stem cell-based therapies in ART relies on their ability to (i) differentiate into reproductive cell types—for example, inducing embryonic stem cells or induced pluripotent stem cells to form oocytes or sperm; (ii) secrete growth factors—stem cells release paracrine signals that enhance tissue repair and cellular function; (iii) integrate into host tissues—transplanted stem cells can integrate into reproductive tissues, contributing to structural and functional recovery.

In the context of ART, stem cells hold potential in several key areas:

Age-related decline in the ovarian reserve is a major cause of infertility. Mesenchymal stem cells and bone marrow-derived stem cells have shown promise in **regenerating ovarian tissue**, improving folliculogenesis, and restoring hormonal balance.^107,108^

Stem cell transplantation has demonstrated potential in **restoring spermatogenesis** in individuals with azoospermia or other forms of male infertility. Spermatogonial stem cells (SSCs) can be harvested, cultured, and reintroduced into the testes to reinitiate sperm production.^109,110^

Conditions such as Asherman’s syndrome and thin endometrium pose significant challenges for successful implantation.^111,112^ Endometrial stem cells (ESCs) and MSCs have been explored to **regenerate and enhance endometrial receptivity**.^113,114^

Advanced research has focused on deriving gametes (eggs and sperm) from pluripotent stem cells. ***In vitro*gametogenesis (IVG)** represents a potential breakthrough for individuals with nonfunctional or absent gametes, offering a new route to biological parenthood.

Various stem cell types used in ART^101^ are exemplified In Table 2.

**Table 2:** Exemplary Stem Cell Types Applied in ART

Stem cells type	Organ	Applied to	Effect/Stimulation
Adipose tissue-derived (ADSC)	Testis	Rat	Spermatogenesis^115^
	Ovary	Mouse, rat	Follicles, estradiol^116^
Umbilical cord (UCSC)	Testis	Mouse	Germ cells^117^
	Endometrium	Human	Endometrium, birth^118^
Induced pluripotent (iPSC)	Testis	Human	Spermatogenesis^119^
	Ovary	Human	Oocytes^120^
Spermatogonial (SSC)	Testis	Macaque	Spermatogenesis^121^
Oogonial (OSC)	Ovary	Mouse	Oocytes, birth^122,123^
Amniotic fluid (AFSC)	Ovary	Mouse	Follicles^124^
Bone marrow (BMSC)	Ovary	Mouse	Follicles^125^
Embryonic (ESC)	Ovary	Mouse	Oocytes^122,126^
Endometrial progenitor cells (EPC)	Endometrium	Mouse	Endometrium, birth^127^

### Advancements in Genetic Screening

One of the most significant trends in ART is the integration of advanced genetic screening techniques. Preimplantation genetic testing (PGT) has become increasingly sophisticated, allowing for the detection of chromosomal abnormalities and single-gene disorders in embryos before implantation.^128,129^

- PGT-A (aneuploidy screening) technique screens for chromosomal abnormalities, which are a leading cause of implantation failure and miscarriage. Advances in next-generation sequencing (NGS) have improved the accuracy and efficiency of PGT-A, leading to higher success rates in IVF cycles.^130,131^

- PGT-M (monogenic disorder) is used to identify embryos carrying specific genetic mutations, enabling couples with hereditary conditions to have healthy offspring. The development of CRISPR-Cas9 and other gene-editing tools has further enhanced the potential for correcting genetic defects at the embryonic stage.^132,133^

- PGT-SR (structural rearrangements) form of testing is designed for individuals with chromosomal translocations or inversions, helping to identify embryos with balanced chromosomal structures.^134,135^

These advancements not only improve the likelihood of a successful pregnancy but also reduce the risk of passing on genetic disorders, offering a more personalized approach to reproductive medicine.

### Gene Editing

With advancements in genetic technologies, particularly gene editing, the landscape of ART is evolving to address not only infertility but also the prevention of genetic diseases. Gene editing in ART holds the promise of reducing heritable disorders, improving embryo selection, and enhancing reproductive success rates. It allows the precise editing of genes in embryos, eggs, or sperm. The CRISPR-Cas9 system is the most prominent tool in gene editing,^136^ enabling precise modifications in the genome. By targeting specific DNA sequences, CRISPR-Cas9 can add, delete, or alter genes, making it a valuable technology in addressing inherited genetic disorders in embryos created via *in vitro* fertilization (IVF).^137,138^

Gene editing can correct mutations in embryos associated with hereditary diseases such as cystic fibrosis, sickle cell anemia, and Huntington’s disease, preventing their transmission to future generations. Another line of application of gene editing in ART is for enhancement of embryo selection—genetic screening combined with editing can improve embryo quality by selecting embryos with the highest potential for successful implantation and development. Gene editing can help also **addressing infertility**—it may help identify and correct genetic causes of infertility, such as chromosomal abnormalities or mutations affecting gamete function.

The technique is controversial due to the potential for “designer babies” and unintended consequences. Moreover, editing one gene could have unforeseen effects on other genes or biological processes, potentially causing harm. Currently gene editing is banned for reproductive purposes in many countries.^139^ Research is ongoing, but clinical use for reproductive purposes remains highly regulated. One of the major safety concern regarding gene editing is the lack of sufficient data on long-term consequences and possible off-target effects.^100^ Unintended mutations could have serious consequences for individuals and future generations making it crucial to fully understand the potential long-term effects before clinical application.^100,140^ Even highly precise gene editing tools like CRISPR can sometimes make edits at unintended locations in the genome (“off-target effects”), which could lead to unforeseen health complications.^141,142^ Regulatory agencies are increasingly engaging with the scientific community to establish frameworks for the safe and ethical use of gene-editing technologies. There is a rising urgency to involve the public in debates regarding the ethical, legal, and social implications of gene editing.

### Mitochondrial Replacement Therapies

Mitochondrial replacement therapy (MRT), also known as mitochondrial donation, is a technique that aims to prevent the transmission of mitochondrial DNA (mtDNA) disorders from mother to child.^143–146^ This involves replacing defective mitochondria in an egg or embryo with healthy mitochondria from a donor, preventing mitochondrial diseases in offspring. Thus, MRT has been used to create embryos with genetic material from three individuals: the mother, the father, and a mitochondrial donor (so-called “three-parent babies”).^147^ MRT raises ethical concerns related to genetic modification and its long-term effects on future generations. MRT is particularly beneficial for women with mitochondrial disorders who wish to have genetically related children. The technology is currently regulated differently across countries, with some permitting its use under strict guidelines and others banning it outright.^148^

Gene editing and MRT technologies are compared in Table 3.

**Table 3:** Comparison of Gene Editing and MRT

Feature	Gene editing	Mitochondrial replacement therapy
Target	Nuclear DNA	Mitochondrial DNA
Scope	Broad (diseases, traits, viability)	Specific (mitochondrial diseases only)
Ethical concerns	Designer babies, germline edits	Three-parent babies, identity concerns
Techniques	CRISPR, base editing, prime editing	PNT, MST, PBT^a^

a PNT, pronuclear transfer; MST, maternal spindle transfer; PBT, polar body transfer.

#### Intracytoplasmic Sperm Injection

Intracytoplasmic sperm injection (ICSI) is an ART procedure that involves injecting live sperm directly into the cytoplasm of a mature egg using a micromanipulation tool. The fertilized egg is then cultured and transferred as in IVF It represents a refinement of IVF and is the most common and successful treatment for male infertility caused by sperm issues, such as low sperm count, poor motility, or abnormal morphology, or when previous IVF attempts have failed. Success rates are similar to IVF, but ICSI can significantly improve fertilization rates in cases of male infertility.^149^

#### Improved Embryo Culture Systems

There are many ways to improve embryo culture systems in ART. These include new culture platform design creating a better microenvironment for embryos, new media formulations including antioxidants to reduce oxidative damage and improve blastocyst development, and perfusion-based systems using dynamic media flow instead of static culture. Advances in time-lapse imaging and monitoring,^150,151^ and optimized culture media^152^ allow continuous monitoring of embryo development, enabling better selection for transfer and increasing implantation rates.

#### Next-Generation Sequencing

Next-generation sequencing (NGS) is a genomic testing technology that is used in ART to screen embryos for genetic defects. Preimplantation genetic testing (PGT) using NGS helps to identify genetic abnormalities in embryos. It can identify euploidy, aneuploidy, and chromosomal mosaicism.^153^ Using PGT enhances the likelihood of healthy pregnancies while minimizing the risk of genetic disorders.^154,155^

#### Cryopreservation Enhancements

Cryopreservation techniques in ART have improved in several ways, including vitrification—a rapid freezing process that prevents ice crystal formation, improving survival rates of frozen gametes and embryos, coupled with improved and optimized cryoprotectants, vapor tanks storing tissue in the vapor phase of nitrogen instead of immersing it in liquid nitrogen, offering better survival rates for frozen eggs, sperm, and embryos, increasing ART success rates.^67,156,157^

#### Fertility Preservation Innovations

Techniques such as ovarian tissue cryopreservation and artificial ovary development are advancing, benefiting individuals facing fertility-affecting medical treatments. Ovarian rejuvenation technique is used to stimulate the ovaries to produce new eggs, particularly in women with diminished ovarian reserve or premature ovarian failure. It may include injecting platelet-rich plasma (PRP) into the ovaries to stimulate tissue repair and egg production or stem cells to regenerate ovarian tissue. The technique is still experimental, with mixed results in early studies.^158–160^

#### Noninvasive and Automated Technologies

(i) Time-lapse imaging—continuous monitoring of embryos without the need for manual handling improves embryo selection and reduces stress on the embryos; (ii) Automated IVF systems—robotics and automation are being integrated into laboratories to improve the efficiency and consistency of processes like fertilization and embryo transfer; (iii) Noninvasive genetic testing—techniques to assess the genetic health of embryos using culture media, rather than invasive biopsy, are being developed to minimize risks.^6,161,162^

#### Artificial Wombs

Research into ectogenesis, or artificial womb technology, aims to support the development of embryos outside the human body. Such technology is providing solutions for individuals unable to carry pregnancies due to medical or anatomical reasons, and advancing neonatal care by supporting extremely premature infants.^163,164^

## Insights from CAS Content Collection Data Survey

We examined the assortment of ART-associated concepts in the published documents (journal articles and patents) in the CAS Content Collection (Figure 3).

**Figure 3:**
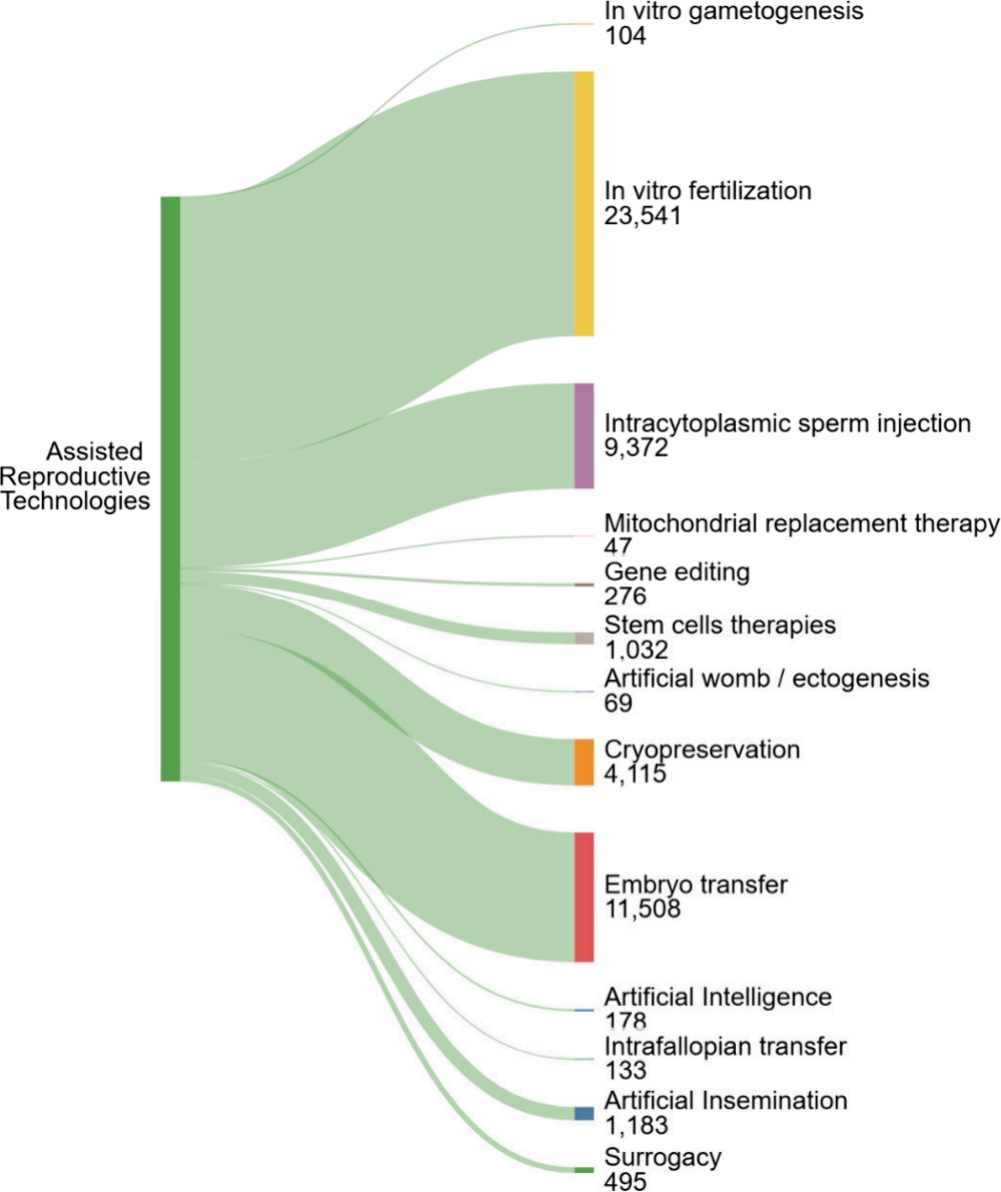
Key concepts related to assisted reproductive technologies in CAS Content Collection with respective numbers of documents for the period 2000–2024.

### In Vitro Fertilization and Embryo Transfer Constitute the Largest Part of ART-Related Documents

Traditional technologies such as *in vitro* **fertilization** and **embryo transfer**, providing major advantages such as proven track record, including successful customization, as well as wide availability, understandably constitute the largest part of ART-related documents in CAS Content Collection^16^ (Figure 3).

Figure 4 illustrates the recent growth (years 2022–2024) and the patent/journal proportions for some of the major ART-related concepts.

**Figure 4:**
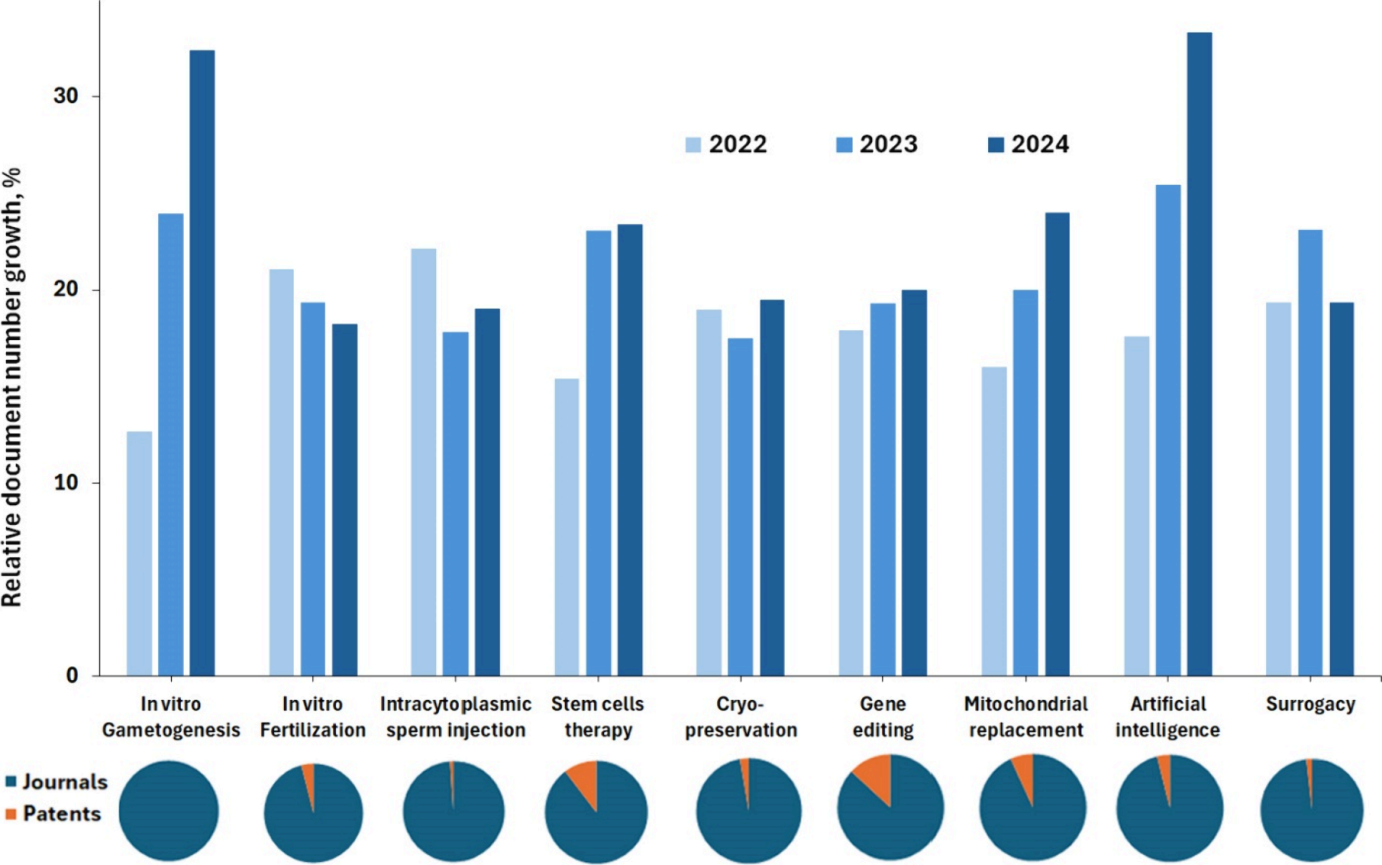
Relative growth of documents associated with the key concepts related to ART in CAS Content Collection over the past 3 years (2022–2024) (top panel stacked bars) and relative proportions of journal articles and patents (bottom row pie charts).

### Artificial Intelligence Integration and in Vitro Gametogenesis Are the Fastest Growing Novel Methods in ART

As seen in Figure 4, artificial intelligence and *in vitro* gametogenesis are the fastest growing novel methods in ART in the last three years (2022–2024).

AI is being used to enhance embryo selection and optimize culture conditions, leading to improved success rates. Machine learning helps identify patterns in embryo development and patient responses, enabling personalized treatment plans. Indeed, notable improvements were observed in the accuracy of diagnosing and predicting successful outcomes in fertility treatments. AI-driven models provided more precise forecasts of the optimal timing for clinical interventions such as egg retrieval and embryo transfer, which are critical to the success of ART cycles.^72–76,78,161,165,166^

*In vitro* gametogenesis offers several potential advantages, including: enabling reproduction for individuals with impaired fertility due to lack of functional sperm or eggs, allowing same-sex couples to have genetically related offspring, providing greater control over genetic selection through embryo screening, and potentially reducing the physical burden on women by eliminating the need for ovarian stimulation during egg retrieval; however, this technology is still in early stages and raises ethical concerns regarding genetic manipulation and potential misuse.^167,168^ IVG has shown promise in animal models, including creating offspring with biological contributions from same-sex parents. While not yet ready for clinical use, it could revolutionize infertility treatments in the future.

Other methods exhibiting substantial growth in the last three years include **mitochondrial replacement** and **stem cells therapies** (Figure 4). The relative number of documents associated with **gene editing** methods also increased (Figure 4).

### Stem Cell-Based Therapies and Gene Editing Are the Methods with Highest Patent/Journal Ratio, Indicative for High Market Interest

As seen from Figure 4, bottom row, gene editing and stem-cell-based therapies are the methods with highest patent fraction (13% and 10%, respectively) of all documents, which is indicative for high market interest.

Stem cell-based therapies in ART offer potential advantages like improving ovarian reserve function, stimulating follicle development, repairing damaged reproductive tissues, and potentially generating new germ cells, potentially providing hope for individuals struggling with infertility due to conditions like premature ovarian failure or low sperm count by leveraging the unique ability of stem cells to proliferate and differentiate into specialized cell types. They represent a cutting-edge approach to address infertility and enhance reproductive health. These therapies leverage the regenerative potential of stem cells to create gametes, repair reproductive tissues, and improve ART outcomes.^169,170^

Gene therapies in ART offer the potential to prevent genetic diseases in future generations by allowing for the identification and correction of genetic mutations in embryos, potentially leading to healthier babies with a reduced risk of inheriting genetic disorders while also providing more options for couples facing infertility due to genetic issues; however, ethical concerns and the need for further research remain significant challenges. Although in its early stages, gene editing is being explored to address infertility caused by genetic mutations. This could also potentially correct genetic issues in embryos before implantation.^171,172^

Currently, gene editing in humans, particularly germline editing (which affects eggs, sperm, or embryos and can be passed on to future generations), is heavily restricted or banned in many countries due to ethical, safety, and societal concerns. Indeed, changes made to germline cells are heritable, meaning that they affect future generations. This raises ethical questions about consent, as future generations cannot consent to these modifications.^140,173–175^ There are fears that gene editing could be used for nontherapeutic enhancements (e.g., selecting for intelligence, appearance, or athletic ability), leading to societal inequality and eugenics-like practices.^176,177^ Also, some groups argue that altering human DNA is “playing God” or interferes with natural processes. There are also safety concerns that current gene-editing technologies, such as CRISPR-Cas9, are not 100% precise and can cause unintended mutations, which could lead to cancer or other health issues. Editing one gene could have unforeseen effects on other genes or biological processes, potentially causing harm. Furthermore, there is no global agreement on how gene editing should be regulated, leading to a patchwork of laws and guidelines. Access to gene-editing technologies could exacerbate existing inequalities, with only wealthy individuals or countries benefiting.

The ban on germline editing in humans remains largely in place globally, with most countries prioritizing caution and ethical considerations. However, the rapid pace of technological advancement and the potential for misuse have highlighted the need for stronger international cooperation and oversight. While somatic cell editing continues to advance and show promise for treating diseases, the debate over germline editing is far from settled with ongoing discussions about its ethical, social, and scientific implications.

## Certain Novel and Emerging ART in Humans Are Still Highly Experimental

It is worth noting that ART is a rapidly advancing field, but the application of certain novel and emerging technologies in humans is still highly experimental, tightly regulated, and surrounded by ethical and legal challenges. We further overview the current status of animal models related to ART. Certain key assisted reproductive technologies applied in animals are summarized in the Supporting Information.

## Animal Models in Assisted Reproductive Technologies Research

The development and optimization of assisted reproductive technologies rely heavily on animal models, which offer valuable insights into reproductive biology and the effects of various ART interventions. Animal models are indispensable in ART research due to their biological and physiological similarities to humans and their role in studying species-specific reproductive processes. Moreover, animal models provide a controlled environment to study the mechanisms of reproduction, test new technologies, and assess the safety and efficacy of ART interventions. Thus, animals enable repeated experiments, ensuring consistent data collection; animal research minimizes direct experimentation on humans in the initial stages of ART development; certain animal species closely resemble human reproductive physiology, making them ideal for translational research; and models help optimize ART for wildlife and livestock with unique reproductive traits. Furthermore, animal models allow for iterative refinement of techniques, provide insights into developmental biology and long-term effects, and enable high throughput testing of interventions.^178–180^

The development and application of ART in humans and model animals follow parallel tracks, with most techniques undergoing extensive testing in animals before being adapted for human use.

Common animal models in ART research include: (i) rodents (mice and rats)—due to their short reproductive cycles, ease of genetic manipulation, and low cost; (ii) livestock (cattle, sheep, and goats)—contributing to both agricultural efficiency and wildlife conservation by adapting techniques for endangered species; (iii) nonhuman primates—the closest models to humans in reproductive biology; (iv) zebrafish—a unique model for early embryogenesis due to their external fertilization and transparent embryos; and (v) wildlife models—supporting global conservation efforts by enhancing genetic diversity and population recovery. Techniques applied to animal models vs humans are compared in Table 4.

**Table 4:** Comparison of Techniques: Model Animals vs Humans

Technique	Model animals	Humans
IVF and ICSI^a^	Fully established; optimized in animals	Widely used clinically
Gene editing	Commonly used for research and testing	Limited to research; no clinical germline use
Cryopreservation	Well-studied in animals	Routinely used clinically
Mitochondrial replacement	Tested extensively in animals	Approved in select countries for clinical use
Artificial wombs	Successful in sheep	Preclinical stage; no human applications
IVG (stem cell gametes)	Functional gametes achieved in mice	Not yet applicable

a IVF, *in vitro* fertilization; ICSI, intracytoplasmic sperm injection; IVG, *in vitro* gametogenesis.

Animal models remain indispensable in ART research, serving as a bridge to ensure that human applications are safe and effective. Certain key assisted reproductive technologies applied in animals are summarized in the Supporting Information.

## Application of Emerging ART in Humans

Although the application of particular ART in humans is still highly experimental, tightly regulated, and surrounded by ethical and legal challenges, certain ART methods are already widely available. While established techniques, such as *in vitro* fertilization (IVF), cryopreservation, egg and sperm donation, and surrogacy are widely used to address infertility and help individuals or couples conceive, emerging technologies are expanding the boundaries of what is possible. Some examples include: (i) time-lapse imaging – advanced embryo monitoring systems improving the selection of viable embryos for transfer; (ii) preimplantation genetic testing screening embryos for chromosomal abnormalities or inherited conditions, reducing the risk of miscarriage and genetic disorders; (iii) in vitro maturation enables immature eggs to mature outside the body, providing an alternative for patients who cannot undergo traditional stimulation protocols; (iv) laser-assisted hatching technique helps embryos implant by softening the protective shell (zona pellucida), which can sometimes hinder implantation in older women or those using frozen embryos; (v) AI is enhancing embryo selection, predicting treatment outcomes, and customizing patient protocols; machine learning models analyze patient data to predict the probability of successful pregnancy, tailoring treatment protocols accordingly. Success rates for ART vary based on factors such as age, the cause of infertility, and the type of procedure. Advanced techniques like genetic testing and AI are helping to improve outcomes.^181–187^

A concise summary of ART success rates by technique, age group, and indication, based on recent data (CDC/SART/ESHRE 2022–2023 reports^188–194^ ) is presented in Table 5. Success rates are measured by live birth per cycle/transfer.

**Table 5:** ART Success Rates by Technique, Age Group, and Indication^a^

Technique	Age group	Indication (common causes)	Success rate (live birth/transfer)	Key factors affecting success
IVF (standard)	<35	Tubal factor, unexplained	50–60%	Embryo quality, ovarian reserve
	35–37	Diminished ovarian reserve	40–45%	Egg quantity/quality
	38–40	Age-related infertility	25–30%	Higher aneuploidy risk
	>40	Severe DOR, advanced age	10–15%	Often requires donor eggs
ICSI	<35	Male factor (low sperm count)	45–55%	Sperm DNA fragmentation
	>35	Combined male/female factors	30–40%	Age impacts egg/sperm synergy
PGT-A (tested IVF)	<35	Genetic risk, recurrent loss	60–70%	Euploid embryo selection
	>35	Aneuploidy prevention	40–50%	Reduced miscarriage risk
Frozen Embryo Transfer (FET)	all ages	Elective freeze-all cycles	50–65%	Better endometrial prep
Donor Egg IVF	any age	Premature ovarian failure	50–60% (per transfer)	Donor egg age (∼25–30 yrs)
Mild/Natural Cycle IVF	<35	Low responders, cost concerns	20–30%	Fewer eggs retrieved

a CDC, Center for Disease Control and Prevention, USA;^195^ SART, Society for Assisted Reproductive Technologies, USA;^188^ ESHRE, European Society of Human Reproduction and Embryology;^190^ DOR, Diminished Ovarian Reserve.

### Key Notes

- Age impact: Success drops sharply after 35 due to egg quality decline (aneuploidy rates: ∼30% at 35, ∼80% at 42).
- ICSI vs IVF: ICSI improves fertilization in male infertility but does not boost live births if sperm is normal.
- PGT-A benefit: Highest in women >35 (reduces miscarriage risk by screening abnormal embryos).
- FET advantage: Frozen transfers often outperform fresh (better hormone synchronization).

Statistical analysis of certain aspects of the emerging trends in ART, synthesizing global data (2018–2023) from registries (SART/ESHRE/ICMART),^196,197^ and market reports are presented below:

#### Growth of ART Procedures Worldwide

Global IVF cycles/year have increased from 1.5 M (2010) to ∼3.2 M (2023) (CAGR: 7.1%); Success rates increase—45% (2023) live birth/cycle (women < 35) vs 32% (2010) due to PGT-A/IVF-ICSI; cost reduction: AI/automation cut lab costs by 18–22% (2020–2023).

#### Age Demographics and Success Rates

Live birth rates have increased in 2022 vs 2015 by 8% for women < 35, by 6% for age of 35–37, by 4% for age of 38–40, and is stable at 12.1% for women > 40.

## Notable Recent Patents

ART-related patents in the CAS Content Collection grow not only in numbers but also in formulation and methodology diversity. Summarized in Table 6 are notable recent patents related to ART, illustrating their diversity.

**Table 6:** Notable Recent Patent Application Publications Related to ART Extracted from the CAS Content Collection

Patent number|Patent Assignee|Publication year	Title	Key features
US20250006297|Cornell Univ.|2025	Predicting embryo ploidy status using time-lapse images	Methods of noninvasively predicting ploidy status of an embryo by receiving a data set with video including a plurality of image frames of the embryo, analyzing them by machine and/or deep learning model, and generating an output prediction of the ploidy status of the embryo
WO2024211701|Univfy Inc.|2024	System and method for creating a quantifiable IVF phenotype map to drive discovery of IVF prognostics	Computer-aided methods for assessing the probability of a patient having an IVF failure, or the probability of the patient having an intermediate IVF treatment outcome
WO2024206465|Eastern Virginia Medical School|2024	mRNA therapeutics for oocyte maturation	Synthetic mRNA coding region encoding a protein involved in oocyte maturation, and a methods of using the mRNA in oocyte maturation or in vitro fertilization
WO2024155955|Emory Univ.; Case Western Reserve Univ.|2024	Indole, derivatives, and uses in reproductive medicine for isolating an oocyte or ovum in use in in vitro fertilization	Methods of isolating an oocyte or ovum for use in in vitro fertilization including contacting a sample comprising an oocyte or ovum with indole, as well as compositions for preserving or culturing oocytes or ova for further use in reproductive medicine
WO2024188292|Taipei Medical Univ.|2024	Method for predicting success rate of pregnancy in infertility treatment	A method for predicting success rate of pregnancy in infertility treatment, including detecting methylation levels of certain genes in a cervical sample from a female subject and determining the success rate of its pregnancy in the infertility treatment based on the result of the methylation levels of the specific genes
WO2024258838|Colossal Biosciences|2024	Method of increasing the efficiency of laser-assisted in vitro fertilization in an animal thereof	A method of increasing the efficiency of laser-assisted in vitro fertilization in an animal by drilling a hole in the zona pellucida of the oocyte with a laser, after obtaining an oocyte and sperm from the animal, maturing oocyte, removing cumulus cells from the oocyte, contacting and incubating the oocyte with the sperm, and allowing for the in vitro fertilization, with the efficiency increased by laser drilling a hole in the zona pellucida of the oocyte
KR2024072320|LG Chem Ltd.|2024	Pharmaceutical composition for promoting implantation of in vitro fertilized embryo	A composition for promoting implantation of ex vivo fertilized embryo with including human chorionic gonadotropin (hCG) as an active ingredient
CN117778604|Beijing Germountx Health Tech. Co.|2024	Gut microbiota markers for predicting pregnancy outcomes with assisted reproductive technology and their applications	Gut microbiota markers in the intestinal flora for predicting pregnancy outcomes with assisted reproductive technol., as a safe, noninvasive, and accurate prediction method. The intestinal flora marker used to predict the pregnancy outcome of assisted reproductive technol. includes Fusobacterium
JP2024025412|Fujita Academy|2024	Method for testing chromosomal aneuploidy of embryo using noncoding RNA for infertility treatment	A method for testing chromosomal aneuploidy in embryos cultured in vitro using a noncoding RNA marker, creating an extracellular RNA profile, generation a learning data of associating the extracellular RNA profile by performing machine learning and determining the presence or absence of chromosomal aneuploidy in an embryo cultured in vitro, which is a test target, from the extracellular RNA profile of the test target embryo, using the trained model
RU2813434|FGBOU VO Kurskii Gosudarstvennyi Meditsinskii Universitet|2024	Prediction of outcomes of in vitro fertilization and embryo transfer programs based on concentrations of erythrocytes and hemoglobin in blood	Methods for prediction the efficiency of in vitro fertilization (IVF) and embryo transfer (ET) programs involving data of general blood anal. The invention provides higher prognostic accuracy of outcomes of IVF and ET programs

## Challenges and Ethical Considerations

While ART hold immense promise, they come with certain challenges and ethical concerns.^182,198^ Ensuring the health of both parents and their offspring is paramount. Therefore, safety and efficacy need extensive validation before clinical application.^69,76,137,199–201^

### Embryo-Related Ethics, Religious and Cultural Perspectives

Creation and disposal of embryos: Creating more embryos than needed raises concerns about what happens to unused embryos. Some view the disposal of embryos as ethically problematic, particularly in cultures or religions that ascribe moral status to embryos. The Vatican’s Donum Vitae (1987) and Dignitas Personae (2008) declare embryo destruction morally equivalent to abortion, as life begins at conception.^202,203^ Furthermore, many conservative Protestant and Islamic scholars equate embryo disposal with “taking a life”, citing Qur’anic versus (e.g., Surah Al-An’am 6:151) and biblical texts (e.g., Jeremiah 1:5).^204^ Moreover, certain philosophers argue embryos are “persons” with moral rights.^205,206^ “Sanctity of Life” vs “Quality of Life”: The former views embryos as inviolable; the latter prioritizes parental autonomy and medical utility.^207^

Other embryo-related ethics issue include also: (i) Embryo selection: preimplantation genetic testing allows the selection of embryos free from genetic disorders but raises concerns about eugenics and the potential for “designer babies”; (ii) Cryopreservation: Long-term storage raises questions about legal ownership and ethical obligations to unused embryos; (iii) Embryonic research: The use of embryos in stem cell research is controversial, with some arguing it violates the sanctity of life.^208–210^

### Parent and Child Rights

There are certain issues related to parentage and identity concerns: (i) Third-party involvement: Use of donors (egg, sperm) and surrogates introduces legal and emotional complexities regarding parental rights and the child’s right to know their genetic origins; (ii) Posthumous reproduction: Using gametes or embryos from deceased individuals raises questions about consent and the welfare of the resulting child; (iii) Legal parenthood: Surrogacy and gamete donation complicate legal definitions of parenthood, leading to custody disputes; (iv) Donor anonymity vs right to know: Should children conceived via donor gametes have access to their biological parents? (v) Psychological effects: Children born via ART may experience identity struggles if their biological and social parents differ; (vi) Same-sex couples and single parents: Societal biases and legal hurdles may affect the access of same-sex couples or single individuals to ART.^182,211–214^

### Genetic and Technological Ethics

Ethical issues related to genetic engineering include: (i) Gene editing: Technologies like CRISPR used in ART raise concerns about unintended consequences, heritable changes, and societal implications of altering human genetics; (ii) Artificial gametes and wombs: The creation of gametes from stem cells and the development of artificial wombs challenge traditional views of reproduction and may blur ethical boundaries; (iii) Germline editing: CRISPR-Cas9 allows heritable genetic modifications, raising fears of eugenics and unintended consequences; (iv) Nonmedical enhancements: Ethical concerns arise if gene editing is used for cosmetic traits (e.g., height, intelligence) rather than disease prevention; (v) Regulation and oversight: How should society balance scientific progress with ethical boundaries? ^198,215–217^

### Social and Cultural Implications

There are serious ethical issues related to commercialization and exploitation of ART: (i) Commodification of reproduction: ART commercialization may lead to exploitation, particularly of egg donors and surrogates, in countries with less regulatory oversight; (ii) Gender and economic inequalities: ART can reinforce inequalities, as wealthier individuals have greater access to advanced treatments; (iii) Population dynamics: Widespread use of ART could influence societal norms regarding family size, age of parenting, and population demographics; (iv) Egg and sperm donation: Financial incentives may exploit economically vulnerable donors; (v) Baby markets: Critics argue that commercializing reproduction commodifies human life; (vi) Global surrogacy industry: Unregulated markets in developing countries raise concerns about coercion and unfair compensation.^218–220^

### Legal and Regulatory Issues

Legal and regulatory issues are another aspect of ethics-related problems in ART: (i) Lack of standardized regulations: ART practices and laws vary widely across countries, leading to ethical inconsistencies; (ii) Cross-border reproductive care (reproductive tourism): People traveling to countries with more lenient ART laws may exploit loopholes, complicating ethical oversight and enforcement; (iii) Privacy and data security: Use of AI and genetic data in ART raises concerns about patient confidentiality and potential misuse of sensitive information.^182,184,221^

### Ethical Issues Related to the Emerging New Trends in ART

Specific ethical issues related to the emerging new trends in ART are summarized in Table 7.

**Table 7:** Ethical Issues Related to the Emerging New Trends in ART

ART technology	Ethical concerns
Mitochondrial replacement therapy (“three-parent babies”)	Genetic modification of future generations: changes are heritable, raising fears of unintended consequences.
	Identity and kinship issues: a child has genetic material from three individuals—how does this affect familial and social identity?
	Safety and long-term effects: unknown risks to offspring and future generations.
In vitro gametogenesis—creating eggs and sperm from stem cells	Designer babies and eugenics: could lead to selection for “desirable” traits.
	Reproductive exploitation: mass production of gametes may commodify reproduction.
	Legal parenthood complications: if gametes can be created from any cell, who is legally the parent?
Artificial wombs (ectogenesis)	Impact on abortion debates: could fetal viability outside the body redefine abortion laws.
	Gender and societal roles: may reduce the biological necessity for women in reproduction, with complex social implications.
	Parent-child bonding: does artificial gestation affect maternal-fetal attachment?
Advanced CRISPR and germline editing	Irreversible changes to the human gene pool: risks of unintended mutations.
	Ethical limits on enhancement: should editing be restricted to medical uses, or could it lead to “designer babies”?
	Global inequality: access may be limited to wealthy individuals, exacerbating social divides.

The World Health Organization (WHO) has called for a global registry of human gene-editing research and stricter oversight.^222,223^ The UNESCO International Bioethics Committee has recommended a moratorium on germline editing.^224–226^ In the United States, germline editing is not explicitly banned but is heavily restricted. Federal funds cannot be used for germline editing research, and the FDA is prohibited from approving clinical trials involving heritable genetic modifications.^227,228^ Many European countries have laws prohibiting germline editing. The Oviedo Convention explicitly bans heritable genome editing.^229,230^ In China, after the controversial case of He Jiankui (who created the first gene-edited babies in 2018), China introduced stricter regulations and penalties for unauthorized gene-editing experiments.^231–233^ The UK allows gene editing in embryos for research purposes but prohibits implantation of edited embryos.^234^ In 2023, the UK approved CRISPR-based therapies for treating blood disorders like sickle cell anemia and beta-thalassemia, marking a significant step forward for somatic gene editing.^235^ The International Summit on Human Genome Editing continues to debate the ethical and scientific implications of germline editing, with many experts calling for a cautious approach. Australia maintains a ban on germline editing, with strict penalties for violations.^236^ However, in 2023, the Australian government began reviewing its gene-editing laws to potentially allow somatic cell editing for therapeutic purposes.^237^

## Conclusion

ART is rapidly evolving with research focused on improving safety, success rates, and accessibility. Future trends involve: (i) tailoring treatments to individual genetic profiles through personalized medicine approach; (ii) improving embryo selection and predicting outcomes via AI integration and automation; (iii) expanded accessibility by developing lower-cost methods to reach underserved populations; as well as (iv) exploring the long-term health of ART-conceived children and refining techniques like artificial gametes.

Emerging technologies in ART are pushing the boundaries of reproductive medicine, offering hope to individuals facing infertility while raising profound ethical and societal questions. From AI-driven embryo selection to *in vitro* gametogenesis and gene editing, these advancements promise to redefine parenthood. However, translating these innovations into clinical practice requires careful consideration of safety, accessibility, and ethical implications to ensure equitable and responsible use. Once an ART innovation proves successful in animal models, it progresses to clinical trials in humans, beginning with small, carefully monitored studies. Innovations such as time-lapse imaging, laser-assisted hatching, and AI-driven embryo selection have all transitioned from theory or animal-based research to human use after rigorous validation.

## Supplementary Material



## Notes

Assisted reproductive technologies in animals (PDF)
