# Supplementary material for: Assisted Reproductive Technology: A Ray of Hope for Infertility
Source: ACS Omega. 2025 May 23;10(22):22347–65. doi: 10.1021/acsomega.5c01643 (PMC12163768; doi:10.1021/acsomega.5c01643)
Supplement: Supplementary file 1 [file ao5c01643_si_001.pdf]

Supporting information

Assisted Reproductive Technology: A Ray of Hope for Infertility

Rumiana Tenchov, Qiongqiong Angela Zhou*

CAS, a division of the American Chemical Society, Columbus OH 43210, USA

*Corresponding author: qzhou@cas.org

Assisted reproductive technologies in animals

Reproduction is a cornerstone of species survival and agricultural productivity. However, challenges such as infertility, inbreeding, and biodiversity loss have necessitated the development of assisted reproductive technologies (ART) in animals. Initially developed for livestock breeding, ART has expanded to address conservation and biomedical research. [1-3]

Key assisted reproductive technologies applied in animals

Artificial Insemination involves the collection of semen from a male and its subsequent introduction into a female's reproductive tract. This method is widely used in livestock breeding to optimize genetic traits, improve productivity, and reduce the risk of disease transmission.

In vitro fertilization enables fertilization outside the body, allowing control over genetic selection and embryo quality. Embryos can be transferred into surrogate mothers or cryopreserved for future use.

Embryo transfer allows embryos from genetically superior females to be implanted into surrogate mothers, increasing the reproductive potential of high-value animals.

Cryopreservation – the freezing of gametes, embryos, and reproductive tissues facilitates long-term genetic preservation and global exchange of genetic material.

Somatic cell nuclear transfer (SCNT) involves transferring the nucleus of a somatic cell into an enucleated oocyte, creating a genetic replica. Cloning supports conservation by replicating endangered animals.

Hormonal synchronization and superovulation – techniques such as estrus synchronization and superovulation increase reproductive efficiency by aligning breeding times and enhancing oocyte production.

Gene editing and transgenics – CRISPR-Cas9 and other genome editing tools are being used to modify animal genomes for desirable traits, such as disease resistance or productivity.

Stem cell-derived gametes – *in vitro* gametogenesis (IVG) from stem cells offers potential for fertility restoration and genetic rescue in critically endangered species.

ART in animals has been applied (i) in agriculture and livestock production, to improve genetic traits, such as milk yield, growth rate, and disease resistance, enhancing productivity and sustainability in livestock industries; (ii) in conservation biology, to address challenges in endangered species conservation by preserving genetic material, supporting captive breeding, and combating inbreeding depression; (iii) cross-species surrogacy has emerged as a strategy to sustain populations of species with critically low numbers. It has been applied also in companion animal breeding, to supports breeding programs for pets, particularly for rare or exotic species, ensuring genetic health and diversity.

Thus, ART have transformed animal reproduction, offering unparalleled opportunities to enhance productivity, conserve biodiversity, and address infertility challenges. Ongoing advancements in ART, including genetic engineering and stem cell technologies, promise to further expand its applications.

References

1. Maleki-Hajiagha, A., Shafie, A., Maajani, K., and Amidi, F. (2024) Effect of astaxanthin supplementation on female fertility and reproductive outcomes: a systematic review and meta-analysis of clinical and animal studies. Journal of Ovarian Research 17, 163.
2. Khasanshina, Z. R., Abdullaeva, D. A., and Bogacheva, N. V. (2024) Comparative Characteristic of the Methods of Isolation of Oocytes and Spermatozoa in Laboratory Animals (Review). Bulletin of Experimental Biology and Medicine 177, 686-690.
3. Sato, M., Inada, E., Saitoh, I., Morohoshi, K., and Nakamura, S. (2024) Artificial Insemination as a Possible Convenient Tool to Acquire Genome-Edited Mice via In Vivo Fertilization with Engineered Sperm. BioTech 13, 45.
